# Unlocking Doors without Keys: Activation of Src by Truncated C-terminal Intracellular Receptor Tyrosine Kinases Lacking Tyrosine Kinase Activity

**DOI:** 10.3390/cells3010092

**Published:** 2014-02-14

**Authors:** Belén Mezquita, Pau Mezquita, Montserrat Pau, Jovita Mezquita, Cristóbal Mezquita

**Affiliations:** 1 Laboratori de Genètica Molecular, Universitat de Barcelona, IDIBAPS. Casanova 143, 08036 Barcelona, Spains; E-Mails: belenmezquita@ub.edu (B.M.); montserrat.pau@gmail.com (M.P.); jmezquita@ub.edu (J.M.); 2 Departament de Ciències Bàsiques, Universitat Internacional de Catalunya, Josep Trueta, s/n 08195 Sant Cugat del Vallès, Spain; E-Mail: pmezquita@uic.es

**Keywords:** VEGFR-1, Flt-1, truncated intracellular VEGFR-1, KIT, truncated-KIT

## Abstract

One of the best examples of the renaissance of Src as an open door to cancer has been the demonstration that just five min of Src activation is sufficient for transformation and also for induction and maintenance of cancer stem cells [1]. Many tyrosine kinase receptors, through the binding of their ligands, become the keys that unlock the structure of Src and activate its oncogenic transduction pathways. Furthermore, intracellular isoforms of these receptors, devoid of any tyrosine kinase activity, still retain the ability to unlock Src. This has been shown with a truncated isoform of KIT (tr-KIT) and a truncated isoform of VEGFR-1 (i_21_-VEGFR-1), which are intracellular and require no ligand binding, but are nonetheless able to activate Src and induce cell migration and invasion of cancer cells. Expression of the i_21_-VEGFR-1 is upregulated by the Notch signaling pathway and repressed by miR-200c and retinoic acid in breast cancer cells. Both Notch inhibitors and retinoic acid have been proposed as potential therapies for invasive breast cancer.

## 1. The VEGF Receptor Tyrosine Kinase Family

Three structurally related tyrosine kinase receptors of the vascular endothelial growth factor (VEGF) have been characterized in mammals: VEGFR-1, VEGFR-2 and VEGFR-3 [2]. These receptors consist of a seven immunoglobulin-loop extracellular domain, a transmembrane domain, a juxtamembrane domain, a split tyrosine kinase domain and a C-terminal tail that mediates the interaction of VEGFR with downstream proteins in the signaling pathway. Binding of VEGF at the N-terminal part of the extracellular domain results in the formation of receptor homo- or heterodimers, a step required for its activation. Dimerization of the receptor induces a conformational change of the intracellular kinase domain that exposes the ATP binding site. Following ATP binding, auto or transphosphorylation in the receptor dimer leads to the activation of downstream signal transducer proteins. The activity of the receptors is regulated by internalization and degradation or by dephosphorylation by protein tyrosine phosphatases.

**Figure 1:**
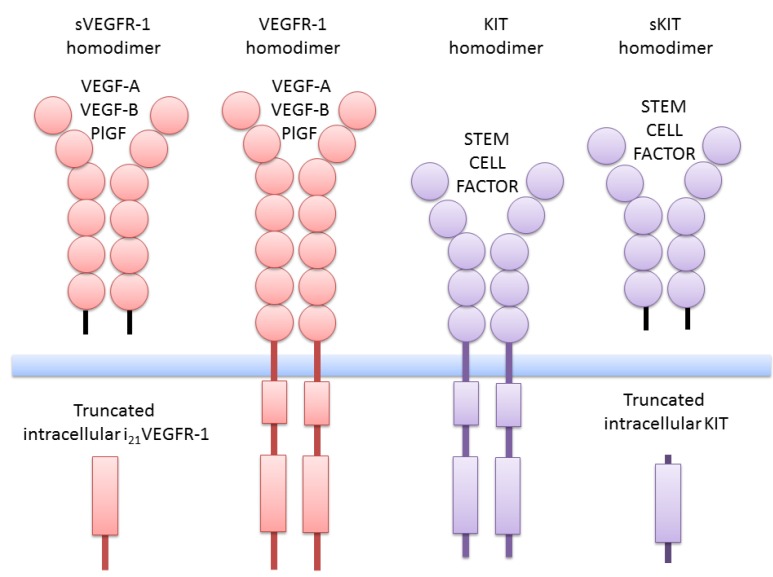
Schematic structure of VEGFR-1 and KIT isoforms. At the center, VEGFR-1 and KIT-homodimer full-length transmembrane receptors. Laterally, extracellular and intracellular truncated isoforms. The intracellular truncated isoforms are the result of alternative transcription initiation in intronic sequences of VEGFR-1 and KIT genes. sVEGFR-1, soluble VEGFR-1; PlGF, Placental Growth Factor; sKIT, soluble KIT.

VEGFR-1 (also known as Flt-1) is a 180–185 kDa glycoprotein [3,4,5] that is activated by VEGF (Figure 1). Three ligands—VEGF-A, VEGF-B and PlGF (Placental Growth Factor)—bind to the immunoglobulin loop 2 of the extracellular domain of VEGFR-1, requiring loops 1 and 3 only to increase binding affinity. The binding affinity of VEGFR-1 is higher by one order of magnitude compared to VEGFR-2, while its tyrosine kinase activity is one order of magnitude lower [6,7,8]. The ligands bind to the receptors in a specific fashion. VEGF-B and PlGF bind selectively to VEGFR-1, whereas VEGF-A binds to VEGFR-1 and VEGFR-2. Binding of VEGF-A induces the formation of receptor heterodimers in VEGFR-1 and VEGFR-2 co-expressing cells [9], in contrast to PlGF or VEGF-B, which are unable to attach to VEGFR-2 [10]. However, binding of PIGF to VEGFR-1 results in the phosphorylation of VEGFR-2 and may sensitize the receptor to subsequent activation by VEGF-A [11].

Overexpression of VEGFR-1 in insect cells or mammalian cells has allowed the identification of several VEGFR-1 tyrosine phosphorylation sites, namely Tyr^794^, Tyr^1169^, Tyr ^1213^, Tyr^1242^, Tyr^1327^ and Tyr^1333^ [5,12,13]. The phosphorylation pattern of VEGFR-1 depends on the ligand. For instance, PlGF, but not VEGFA, induces phosphorylation of Tyr^1309^ [11]. These phosphorylations determine the ability of the receptor to activate different components in the signal transduction. Tyr^794^ [12] and Tyr^1169^ [14] are involved in binding and activation of phospholipase C-γ, whereas Tyr^1213^ binds to SH2-containing proteins [13,15]. VEGFR-1 transduces signals for migration and invasion of cancer cells, via the cytoplasmic tyrosine kinase Src [16,17].

## 2. Truncated Isoforms of VEGFR-1

The VEGFR-1 gene in humans consists of 30 exons spanning more than 193 Kb [10]. One truncated extracellular soluble isoform (sVEGFR-1/sFlt1) is produced using the first 13 exons and an additional sequence located in intron 13 [18]. The sVEGFR1 consists of six immunoglobulin-loops as illustrated in Figure 1. We characterized another transcript that contains the first 14 exons and an additional sequence of intron 14 (s14VEGFR-1), which encodes a protein with a C-terminal polyserine tail (GenBank EU360600). This isoform was reported by Thomas *et al.* [19,20]. Soluble VEGFR-1 can also be obtained by post-translational processing. A truncated extracellular isoform derives from the endoproteolytic cleavage of VEGFR-1 in endothelial cells [21]. Ectodomain shedding of VEGFR-1 has also been observed in leukemic cancer cells [22]. Following the removal of the ectodomain, the remnant of VEGFR-1 remains attached to the membrane and the activity of γ-secretase is required for its release to the cytosol. The soluble forms of VEGFR-1 can modulate the VEGF/VEGFR transduction pathways.

**Figure 2:**
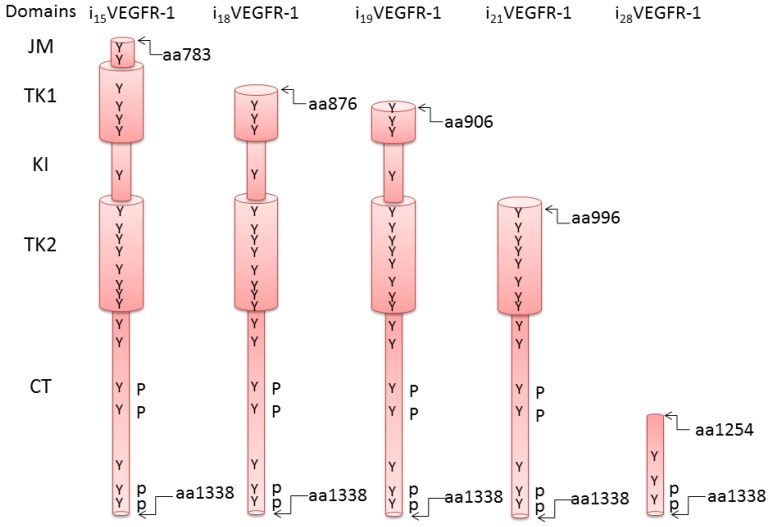
Schematic structure of the intracellular truncated isoforms of VEGFR-1. Amino acid numbers correspond to the full length transmembrane receptor. JM, juxtamembrane domain; TK1, kinase domain, ATP binding; KI, Kinase insert; TK2, kinase domain, phosphotransferase; CT, C-terminal tail region.

We have characterized several transcripts that initiate transcription in intronic sequences of the VEGFR-1 gene [23]. These transcripts have lost the sequences coding for the extracellular domains of the receptor and contain either the full set of intracellular domains or a partial kinase domain followed by the C-terminal sequence (Figure 2). Five transcripts have been identified and named after the intron where transcription initiates (i_15_VEGFR-1, i_18_VEGFR-1, i_19_VEGFR-1, i_21_VEGFR-1 and i_28_VEGFR-1). Additionally, two isoforms (i_15as_VEGFR-1 and i_21as_VEGFR-1) result from alternative splicing of i_15_VEGFR-1 and i_21_VEGFR-1, respectively. All transcripts incorporate additional 5' leader sequences derived from the corresponding 5' intron [23] (GenBank JF509744 and JF509745).

Transcript i21VEGFR-1 is expressed in human endothelial cells, macrophages, fibroblasts, breast cancer MDA-MB-231 cells, and human placenta [23]. The i_21_VEGFR-1 protein is expressed in human endothelial cells and MDA-MB-breast cancer cells [23,24]. The human isoforms i_19_VEGFR-1 and i_28_VEGFR-1 are expressed in human testis (GenBank JF509744 and JF509745). The two i_21_VEGFR-1 transcripts initiate at nucleotide 157 of intron 21. Isoform i_21as_VEGFR-1 putative coding region would start with the specific amino acid MNSDLLV sequence, followed by the whole CDS of exon 22. Putative protein i_21as_VEGFR-1 would have 360 amino acids, and the sequence would be identical to the amino acids 986–1338 (AF063657) of the full-length VEGFR-1 (Figure 2). The protein i_21_VEGFR-1 would contain 343 amino acids, and the sequence would be identical to the amino acids 996–1338 (AF063657) of the full-length VEGFR-1 (Figure 2). These isoforms conserve 163 (i_21_VEGFR-1) and 174 (i_21as_VEGFR-1) of the 332 amino acids of the kinase domain, including none (i_21_VEGFR-1) or 11 amino acids (i_21as_VEGFR-1) of the kinase insert. Both i_21_VEGFR-1 isoforms lack the ATP-binding domain [23]. 

Protein i_21_VEGFR-1 was detected by Western blot analysis [23,24]. To confirm the specificity of the bands detected by the anti-VEGFR-1 antibody, we inhibited the expression of VEGFR-1 and i_21_VEGFR-1 by RNA interference. Bands of 170 kD and 39 Kd, corresponding to the full-length transmembrane VEGFR-1 and the truncated intracellular isoform, respectively, disappear after RNA interference in human endothelial cells (HUVECs). Furthermore, the band of 39 kD, corresponding to i_21_Flt1, is no longer detectable after RNA interference of i_21_VEGFR-1 in MDA-MB-231 breast cancer cells [24].

## 3. The KIT Receptor Tyrosine Kinase Family

The KIT receptor belongs to the type III group of receptor protein tyrosine kinases, together with the vascular endothelial growth factor receptor (VEGFR), the receptor for platelet-derived growth factor (PDGFR) and the receptor for the granulocyte macrophage colony-stimulating factor-1 (CSGFR) [25,26,27,28]. The KIT full-length transmembrane receptor consists of an extracellular domain composed of five immunoglobulin-like repeats, a transmembrane domain, a juxtamembrane domain, a tyrosine domain divided into two parts by a kinase insert domain, and a C-terminal tail (Figure 1). Binding of the ligand stem factor to the KIT receptor results in dimerization of two receptor monomers, followed by autophosphorylation of specific tyrosine residues and recruitment of signaling proteins to the homodimer. Phosphorylation of the signaling proteins activates several transduction pathways. 

The KIT gene codes for two full-length receptors that result from alternative splicing: KITA and KITB. They differ by the presence (KITA) or the absence (KITB) of the amino acid sequence GNNK in the juxtamembrane region of the extracellular domain. Activation of KITB in a myeloid cell line produces activation of Src rather than the PI3 kinase pathway. KITB, but not KITA, shows constitutive tyrosine phosphorylation when transfected into COS7 cells [29] and it is tumorigenic in nude mice when transfected to NIH3T3 fibroblasts [30].

## 4. Truncated KIT Isoforms

In addition to the full-length transmembrane KIT receptors, there is a truncated extracellular form of KIT (sKIT) consisting almost entirely of the extracellular domains of the full receptor. The soluble form is produced by post-translational proteolytic cleavage of full-length KIT. The proteolytic cleavage generates a truncated intracellular form of 50 kDa that remains attached to the cell membrane [31,32,33]. Both KIT isoforms A and B are susceptible to cleavage. The soluble form can modulate the stem factor/KIT transduction pathway.

The third protein encoded by the KIT gene arises from transcription initiation at KIT intron 15 in humans and intron 16 in rodents [34]. Transcription probably occurs through the use of an alternative cryptic promoter. The truncated intracellular isoform is expressed in postmeiotic male germ cells, hematopoietic stem cells, progenitor cells [35], tumor cell lines and tumors [36]. The truncated intracellular KIT protein is 202 amino acids long and lacks the extracellular domains, transmembrane domain, juxtamembrane domain, ATP binding domain and most of the kinase insert domain of the full-length receptor. It contains just a short sequence of the interkinase segment, the phosphotransferase domain and the C-terminal tail of the receptor. While other truncated tyrosine kinase receptors aberrantly expressed in cancer are constitutively active kinases, truncated c-KIT does not contain the ATP binding domain and should be catalytically inactive. However, despite being devoid of tyrosine kinase activity, truncated KIT is able to activate the Src kinase pathway [37].

## 5. Src Activation through Receptor Tyrosine Kinases and Their Intracellular C-Terminal Truncated Isoforms

The c-SRC non-receptor tyrosine kinase is overexpressed and activated in a large number of human malignancies and has been linked to the development of cancer and progression to distant metastases [38,39,40]. Src (for sarcoma) was the first oncogene discovered, and its protein product was the first identified tyrosine kinase [41]. In 1909, Peyton Rous, a young pathologist working at the Rockefeller Institute in New York, discovered that cell-free filtrates obtained from a spontaneous chicken sarcoma could transmit the disease to other individuals, postulating the hypothesis of viral transmission [42,43]. The relevance of Rous’s discovery was recognized 55 years later, as he received the Nobel Prize of Physiology or Medicine. The viral oncogene (v-Src) was a mutated version of the chicken Src normal gene, which was incorporated into the viral genome by recombination and encoded a protein tyrosine kinase [41]. The discovery of the cellular origin of a viral oncogene suggested that normal genes could become oncogenic if inappropriately activated. 

**Figure 3:**
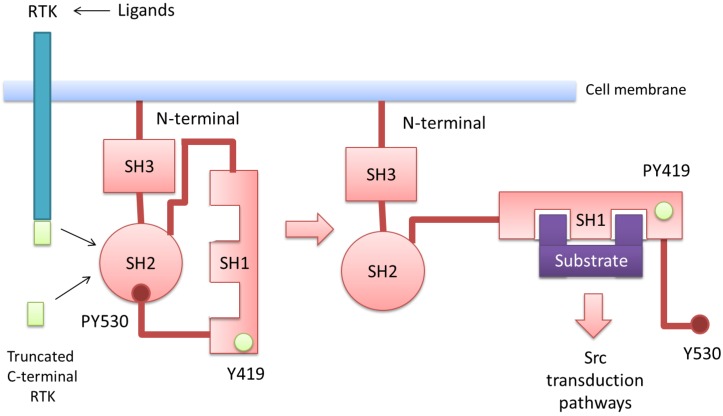
Schematic representation of Src in the low activity state (left) and the active state (right). In the low activity configuration, the SH2 domain binds the phosphorylated C-terminal Tyr^530^, while the SH3 domain interacts with the linker domain, promoting a relative “closed” conformation. In the active configuration, SH2 and SH3 domains are released from the intramolecular interactions with autophosphorylation of Tyr^419^, which enhances the catalytic activity of Src. Activation of Src is mediated by activation of transmembrane tyrosine receptors (RTK) upon binding the corresponding ligands or, alternatively, by the C-terminal intracellular truncated isoforms.

In addition to Src, other members of the family of non-receptor-tyrosine kinases have been characterized: Fyn, Yes, Lyn, Lck, Hck, Blk, Yrk and Fgr [38]. The Src protein is composed of seven domains [38,44,45] (Figure 3): (1) An N-terminal region, which contains a myristoylation sequence, which is essential for binding to the inner surface of the cell membrane. (2) A unique domain that confers specificity to the different members of the Src family. (3) The SH3 domain, which binds to proline sequences and mediates intra and inter-molecular interactions. (4) The SH2 domain, which binds phosphorylated tyrosine residues of the Src molecule itself or other proteins. (5) A linker domain, involved in the intramolecular binding with SH3. (6) A catalytic domain that contains an autophosphorylation site at Tyr^419^, required for maximal kinase activity, and (7) A C-terminal tail, containing the negative-regulatory Tyr^530^.

When Tyr^530^ is phosphorylated, the C-terminal domain binds to the SH2 domain, and Src acquires a closed conformation with low activity. Binding of SH3 to the proline rich linker domain stabilizes this low activity conformation [46,47]. These intramolecular interactions keep the active site of the kinase poorly accessible to substrates [48]. Upon dephosphorylation of Tyr^530^ or displacement of the SH2 and SH3 intramolecular interactions by other proteins, Src-like kinases acquire a relaxed conformation. This allows autophosphorylation of Tyr^419^ in the catalytic site and the achievement of maximal kinase activity [47]. Since the viral oncogenic protein v-Src lacks the regulatory C-terminal tyrosine residue, it is constitutively active and oncogenic.

The tyrosine kinase c-Src interacts physically with multiple tyrosine kinase receptors via its SH2 domain. Src is a substrate of RTKs and, at the same time, an activator of RTKs. This bidirectional activation creates a positive-regulatory loop that contributes to the robustness and persistence of RTK signaling [49]. The binding of RTK with its corresponding ligand leads to receptor dimerization and autophosphorylation on tyrosine residues of the C-terminal tail. Phosphorylated tyrosine residues recruit and activate Src, which then phosphorylates RTK and augments RTK tyrosine kinase activity. The RTK cooperating receptors include epidermal growth factor (EGFR), vascular endothelial growth factor (VEGFR), platelet derived growth factor receptor (PDGFR), fibroblast growth factor receptor (FGFR), insulin-like growth factor receptor (IGFR-1), hepatocyte growth factor receptor (MET), colony stimulating growth factor receptor (CSGFR) and stem factor receptor (KIT), among others. All these receptors and the corresponding ligands are the keys that can unlock the closed structure of Src, opening the door to cancer. In addition, it is also possible to unlock Src without keys, by using the truncated C-terminal isoforms of KIT and VEGFR-1, which lack the ATP binding domain and therefore present no tyrosine kinase activity. Truncated KIT and Src kinase Fyn interact physically through Tyr^161^ of truncated KIT [37]. Upon binding to tr-KIT, Fyn phosphorylates Tyr^161^ of tr-KIT *in vitro* and in transfected cells. A hypothetical model assumes that Fyn, in the low activity conformation, phosphorylates tr-KIT. This phosphorylation allows the interaction of the phosphorylated tr-KIT with the SH2 domain of Fyn, displacing the intramolecular inhibition [37]. Upon relaxation, Fyn autophosphorylates and phosphorylates the proteins involved in the transduction pathway.

**Figure 4:**
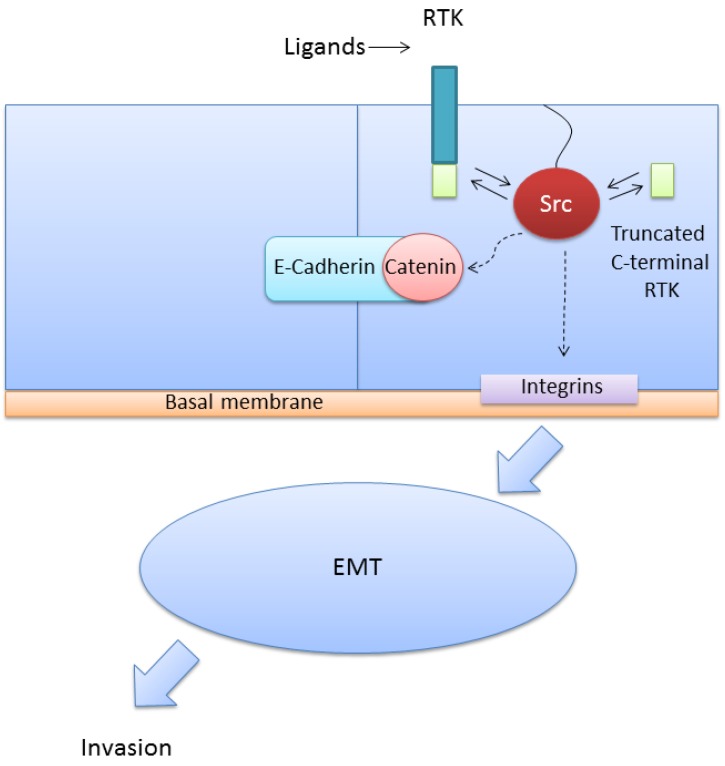
Src signaling pathways and function. Binding of ligands to the corresponding transmembrane tyrosine kinase receptors (RTK) or intracellular truncated C-terminal isoforms of RTKs can activate Src. Activation of Src is involved in different signaling pathways. Particularly important are the disruption of adherens junctions stabilized by E-cadherin and the disruption of focal adhesions, which promotes migration, invasion and metastasis.

The introduction of v-Src into normal cells produces a fully transformed phenotype with simultaneous activation of several transduction pathways such as STAT3, Ras/MAPK and PI3K/AKT. Activation of Src increases migration, invasion and metastasis. Src has a prominent role in invasive migration. Upon activation, Src disrupts adherens junctions between cells stabilized by E-cadherin. Phosphorylation by Src of the E-cadherin-β-catenin complex results in dissociation of β-catenin and functional loss of E-cadherin [50]. Free β-catenin translocates to the nucleus and induces transcription of genes related with the epithelial-mesenchymal transition (EMT). Src activation is a potent trigger for EMT, which is reverted back by inhibition of Src. Activated Src also disrupts focal adhesions that attach cells to the extracellular matrix through integrins. In addition, activated Src promotes the expression of matrix-degrading proteases such as metalloproteinases that enhance the metastatic potential [51] (Figure 4). Src activation also increases angiogenesis, facilitating metastasis formation. Src induces angiogenesis in two different ways: (1) Inducing the expression of angiogenic factors such as VEGF and IL8 [52,53,54] and (2) Cooperating with VEGF receptors [16]. Inhibition of Src can suppress endothelial cell proliferation and migration of human umbilical vein endothelial cells [55].

Recently, Iliopoulos *et al.* [1] reported an interesting experimental model to follow the consequences of Src activation in cancer. A transient activation of Src, as short as 5 min, was sufficient to induce stable neoplastic transformation in immortalized breast epithelial cells. In this model, a spontaneously immortalized cell line, derived from normal mammary epithelial cells, was transfected with ER-Src, a fusion of the Src kinase oncoprotein (v-Src), and the ligand binding domain of the estrogen receptor. When these cells were treated with tamoxifen (TAM), a phenotypic transformation occurred. The transformed cells formed colonies in soft agar, showed increased motility and invasive ability, and tumor formation upon injection in nude mice. Transformed ER-Src cells formed mammospheres, whereas the untransformed cells did not. Strikingly, mammospheres derived from ER-Src-transformed cells could be passaged *in vitro* for 12 generations in the absence of TAM, with the number of mammospheres increasing upon passage. As expected from the absence of TAM, the passaged mammospheres did not contain activated Src (assayed by phosphorylation of Y^419^), unlike the initially transformed cells in the presence of TAM. Remarkably, when TAM treatment lasted only 5 min, stable transformation was produced. A progressive increase of the time of TAM treatment reduced the time required to obtain the transformed phenotype. 

In this model, NF-κB is activated within 30 min after Src activation and remains highly active until 36 h after TAM treatment. Activation of NF-κB rapidly increases the expression of the protein Lin28B and increased levels of Lin28B inhibit the expression of the microRNA let-7 through a posttranscriptional mechanism. Lin28B and its ability to rapidly inhibit let-7 microRNAs upon Src activation is a key early step that is important for cellular transformation. Let-7 microRNA directly inhibits expression of IL6 through binding the 3' UTR of the IL6 mRNA. IL6 is important for transformation. When IL6 was depleted by a monoclonal antibody, the morphological changes associated with transformed cells were blocked, and colony formation and cell motility were inhibited. 

IL6 acts primarily through its receptor to activate the JAK/STAT pathway, and inhibition of the IL6 receptor reduces transformation and tumorigenicity. STAT3, a DNA-binding transcriptional activator that is phosphorylated in response to IL6, is an important mediator of cellular transformation. IL6 inhibition strongly reduces STAT3 expression and phosphorylation, indicating that STAT3 activation is IL6 dependent. IL6 activates NF-κB and activation of NF-κB increases IL6 expression, resulting in a positive feedback loop [1]. The positive feedback loop induced by Src activation, and the resulting transformed phenotype, are maintained in the absence of Src activity. 

The positive feedback loop that produces IL6 is important for cancer cells from diverse developmental lineages. Eight out of 15 different kinds of cancer cell lines show Lin28B overexpression, let-7 downregulation, and high levels of IL6. Perturbation of any component of the regulatory circuit significantly reduced the tumorigenicity and motility of lung (A549), hepatocellular (HepG2), breast (MDA-MB-231), prostate (PC3), and colon (Caco2) cancer cells. In all cases, these perturbations resulted in reduced expression of IL6, suggesting the importance of IL6 in maintaining the transformed phenotype. 

**Figure 5:**
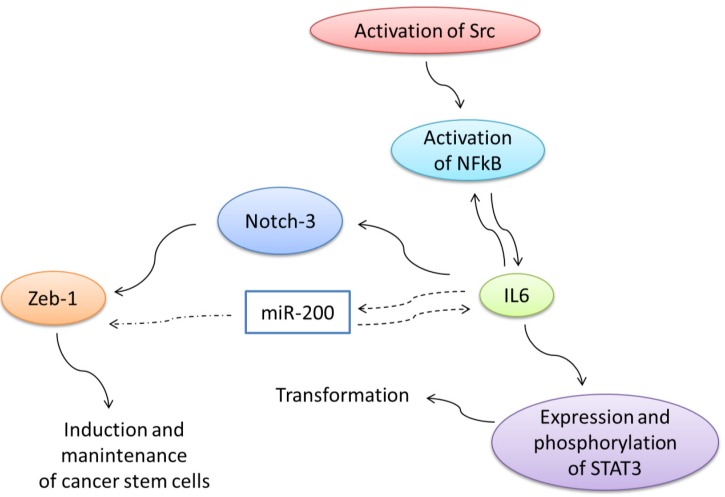
Signaling circuit driving transformation and tumorigenesis. After Src activation, NF-κB is activated and IL6 is produced [1]. STAT3, a DNA-binding transcriptional activator that is phosphorylated in response to IL6, is an important mediator of cellular transformation. IL6 activates NF-κB and activation of NF-κB increases IL6 expression, resulting in a positive feedback loop. In addition to cell transformation, the activation of IL6 expression is important for induction and maintenance of cancer stem cells. IL6 regulates negatively the micro RNA family miR-200 and positively the Notch-3 transduction pathway. Both, downregulation of miR-200 and activation of Notch are important for induction of the epithelial-mesenchymal transition and for induction and maintenance of cancer stem cells.

In addition to cell transformation, the expression of IL6, after Src activation, is important for induction and maintenance of cancer stem cells [56]. IL6 regulates, negatively, the micro RNA family miR-200 and, positively, the Notch-3 transduction pathway. Both downregulation of miR-200 and activation of Notch are important for induction and maintenance of cancer stem cells [57,58] (Figure 5).

## 6. Expression of the Full-Length VEGFR-1 and the Truncated Intracellular Isoforms in Cancer Cells is Related with Increased Migration and Invasion through Activation of Src

Hiratsuka *et al.* [59] originally reported that mouse lacking the tyrosine domain of VEGFR-1 (Flt1^TK−/−^ mice) had impaired metastatic progression. Several reports demonstrated that inhibition of VEGFR-1 by anti-VEGFR-1 peptide blocked micro- and macrometastasis, while overexpression of placental growth factor (PlGF), which signals exclusively through VEGFR-1, increased metastatic spread [60,61]. VEGFR-1 is required for lung adenocarcinoma cell invasion and metastasis [62]. Knocking down VEGFR-1 in lung cancer cells decreased proliferation in monolayer culture, colony formation in soft agar, invasion in coculture with cancer associated fibroblasts, and metastatic potential following subcutaneous injection into syngeneic mice [62]. Similarly, VEGFR-1 maintained cell survival in colorectal and pancreatic cancer cells, and was required for tumor cell migration and invasion [63,64]. VEGFR-1 activation induced tumor cell epithelial-mesenchymal transition and increased cell invasion through phosphorylation of Src family members [16,65,66].

In addition to expression of VEGFR-1 in cancer cells, expression of VEGFR-1 in cells of the tumor microenvironment is also important for metastasis. VEGFR1-positive hematopoietic bone marrow progenitors initiate the pre-metastatic niche [67]. Knockdown of VEGFR-1 in myelomonocytic cells eradicates micro- and macrometastases (see Kaplan *et al.* reply to [68]). Chemotherapy-induced expression of VEGFR-1 on endothelial cells can create an environment favorable to tumor cell homing [69].

The full-length VEGFR-1 receptor and the soluble form (sVEGFR-1) were not detected in MDA-MB-231 by Northern blot analysis of total RNA [23], in accordance with a previous report [70] and were barely detectable by RT-PCR in comparison with a high expression in endothelial cells. Highly invasive MDA-MB-231 breast cancer cells showed epigenetic gene silencing of VEGFR-1 as a consequence of promoter hypermethylation [71]. Aberrant promoter methylation of VEGFR-1 was also reported in prostatic cancer [72] and in 15 cancer cell lines studied [71]. Western blot analysis showed high expression of the full-length VEGFR-1 receptor in endothelial cells but not in MDA-MB-231 cells [23]. Only one band of higher mobility than the corresponding to the full-length VEGFR-1 transcript was detected by Northern blot in MDA-MB-231 cells. This band was identified as the intracellular truncated isoform i_21_VEGFR-1 [23].

The amount of i_21_VEGFR-1, transcript and protein, expressed in MDA-MB-231 cells, varies with cell culture conditions [23]. When MDA-MB-231 culture medium was changed every day, the amount of i_21_VEGFR-1 was barely detectable. However, the amount increased markedly when cells were maintained for 5–6 days without any change of the culture medium, suggesting that a paracrine control may increase expression of i_21_VEGFR-1 [23]. 

Since i_21_VEGFR-1, transcript and protein is the main VEGFR-1 isoform expressed in MDA-MB-231 breast cancer cells, our initial approach to study the function of this isoform consisted in inhibiting its expression by RNA interference or overexpressing the intracellular isoform by transfection of i_21_VEGFR-1 [23]. The ability of MDA-MB-231 siRNA transfected cells to migrate or to invade through Matrigel was substantially decreased after silencing i_21_VEGFR-1 and markedly increased after overexpression of i_21_VEGFR-1, as compared to control cells [23]. 

To determine if Src is activated by the intracellular isoform i_21_VEGFR-1 we performed experiments of interference or overexpression of i_21_VEGFR-1 in MDA-MB-231 cells [23]. Silencing i_21_VEGFR-1 by RNA interference decreases Src phosphorylation at Tyr^419^, as demonstrated by Western blot analysis with a specific antibody against Y^419^-Src peptide. To test further the effect of i_21_VEGFR-1 on Src phosphorylation at Tyr^419^, we transfected MDA-MB-231 cells with i_21_VEGFR-1. Cells stably or transiently transfected with i_21_VEGFR-1 upregulate the active form of Src. Src activation has been implicated in cell invasion and could be a potential mechanism to explain the increase of cell invasiveness produced by i_21_VEGFR-1. Src kinase inhibition by PP2 produces a similar effect to silencing i_21_VEGFR-1, decreasing the capacity of MDA-MB-231 cells to pass through a Matrigel barrier [23].

## 7. Regulation of Expression of Truncated Intracellular VEGFR-1 in Breast Cancer Cells

Expression of i_21_-VEGFR1, transcript and protein in MDA-MB-231 highly invasive breast cancer cells is controlled by the Notch signaling pathway [23,24]. Interference of the Notch signaling pathway by the inhibitor of γ-secretase DAPT decreases the expression of i_21_VEGFR-1 in MDA-MB-231cells [24]. Interference of the Notch-1 and Notch-3 signaling pathways by siRNA downregulates the truncated isoform in these cells [24]. By contrast, activation of Notch signaling *in vitro* by the ligand Dll4 activates the expression of i_21_VEGFR-1 protein in MDA-MB-231 cells [24]. 

A role for the Notch pathway in tumor metastasis has been proposed [73,74,75,76,77]. Since we have reported that i21VEGFR-1 can activate Src and increase the invasiveness of MDA-MB-231 cells [23], Notch-1 and Notch-3 signaling pathways could contribute to the invasive phenotype of MDA-MB-231 breast cancer cells through upregulation of i21VEGFR-1 protein.

In addition to the positive regulation by the Notch pathway, the expression of i21VEGFR-1 is negatively regulated by the micro RNA family miR-200 [24]. VEGFR-1 has been validated as a miR-200s target and overexpression of miR-200s reduced significantly the expression of VEGFR-1 in both lung adenocarcinoma cells [62] and colon cancer cells [78]. Both the full-length receptor and the intracellular truncated i21VEGFR-1 transcript possess the same 3’UTR with the target sequences for miR-200s. The expression of the protein i21VEGFR-1 was markedly reduced in MDA-MB-231 cells transfected with pre-miR-200c [24]. When MDA-MB-231 cells were cultured during six days without changing the culture medium, miR-200c decreased markedly, while i21VEGFR-1 increased [24]. Reexpression of miR-200s in highly invasive MDA-MB-231 breast cancer cells decreased motility and invasion *in vitro* and suppressed pulmonary metastasis *in vivo* [79]. 

**Figure 6:**
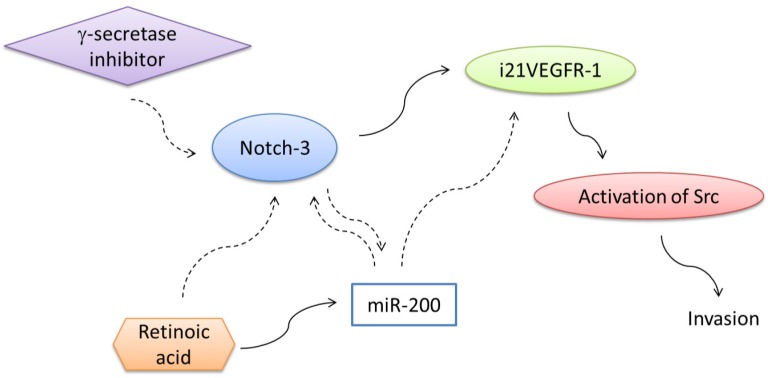
Expression of i21VEGFR-1 is positively regulated by the Notch pathway and negatively regulated by the micro RNA family miR-200. Addition of retinoic acid to the culture medium of MDA-MB-231 breast cancer cells inhibits the expression of the protein i21VEGFR-1. The effect of retinoic acid on the expression of the intracellular truncated isoform of VEGFR-1 is mediated by downregulation of Notch-3 expression. Moreover, retinoic acid increases the expression of the miR-200 family of micro RNAs in MDA-MB-231 breast cancer cells. The expression of the truncated intracellular protein i21VEGFR-1 decreases when the Notch signaling pathway is interfered withγ-secretase inhibitors.

Addition of retinoic acid to the culture medium of MDA-MB-231 breast cancer cells inhibits the expression of the protein i21VEGFR-1 [24]. The effect of retinoic acid on the expression of the intracellular truncated isoform of VEGFR-1 could be mediated by the Notch or/and miR-200 pathways. Retinoic acid does not change the expression of Notch-1 in MDA-MB-231 cells [24]. However, the expression of Notch-3 decreases markedly [24]. This observation is in agreement with a previous finding of downregulation of Notch-3 expression by retinoic acid in MCF7 breast cancer cells [80]. Moreover, retinoic acid increases the expression of the miR-200 family of micro RNAs in MDA-MB-231 breast cancer cells [24]. MDA-MB-231 breast cancer cells treated with retinoic acid showed an increase in the expression of miR-200a, miR-200b and miR-200c [24]. An inverse relationship between miR200 expression and Notch activity has been previously reported in MDA-MB-231 breast cancer cells [81]. Links between γ-secretase inhibitors, retinoic acid, Notch pathway, miR-200 and i21VEGFR-1 are shown in (Figure 6). Both γ-secretase inhibitors and retinoic acid have been proposed as potential therapies for invasive breast cancer. 

## 8. KIT, Truncated Intracellular KIT, and Cancer

As previously indicated, transfection of KITB to NIHT3 fibroblasts is tumorigenic in mice [30]. Mutations of KIT are associated with gastrointestinal stromal tumors, myeloid leukemias and testicular seminomas [82,83]. These mutations induce ligand-independent dimerization and autophosphorylation of KIT and constitutive activation of downstream signaling pathways.

Expression of the intracellular truncated isoform of KIT has been observed in 30% of the gastrointestinal and hematopoietic tumor cell lines studied [84]. Western blot analysis of 23 primary prostate cancers indicated that tr-KIT was expressed in ~28% of the tumors at less advanced stages and in 66% of those at more advanced stages, whereas it was not expressed in benign prostatic hypertrophies [36]. Prostate cancer cell lines and tumors expressing the tr-KIT have higher levels of phosphorylated/activated Src than tr-KIT-negative cells and tumors. Transfection of tr-KIT into prostate cancer cells caused a dramatic increase in Src activity. Sam68, an RNA-binding protein phosphorylated by Src, is phosphorylated only in prostate tumors expressing the tr-KIT. These observations by Paronetto *et al.* [37] showed for the first time the existence of a truncated c-KIT protein in primary tumors and showed a correlation between tr-KIT expression and activation of the Src pathway in the advanced stages of the disease. 

## 9. Structural and Functional Similarities between Truncated Intracellular Isoforms of VEGFR-1 and KIT

Since the intracellular C-terminal truncated isoforms of KIT and i_21_VEGFR-1 are similar in structure, and both activate Src [23,37], it is possible that these proteins share similar functions. The truncated intracellular isoform of KIT is expressed in post-meiotic stages of spermatogenesis [34]. When microinjected into mouse eggs, truncated intracellular KIT causes parthenogenetic activation through activation of Src family kinases, suggesting that it might play a role in fertilization [37]. Two truncated intracellular isoforms of VEGFR-1, i_19_VEGFR-1 and i_28_VEGFR-1, are expressed in human testis (GenBank JF509744 and JF509745). We do not know if i_19_VEGFR-1 and i_28_VEGFR-1 have a similar function to tr-KIT in parthenogenetic activation of eggs. In addition to this function, there is the possibility that the truncated intracellular isoforms of VEGFR-1 and KIT, present in spermatozoa, may activate Src during the process of capacitation, a pre-requisite that allows spermatozoa to gain the ability to fertilize an oocyte. Src activation is critical to promote the tyrosine phosphorylation events associated with human sperm capacitation [85]. 

Another function of i_21_VEGFR-1 and tr-KIT is their capacity to induce cancer cell invasiveness [23]. While the full length transmembrane receptor VEGFR-1 can induce migration and invasion through activation of Src, the truncated C-terminal intracellular isoform i_21_VEGFR-1 is able to activate Src in the absence of tyrosine kinase receptor ligands or the full length transmembrane receptor [23]. Cancer therapies based on the interference of ligands or receptors should consider the possibility of intracellular activation of Src by truncated isoforms. Due to the critical role of transient Src activation in the signaling circuit responsible for transformation and tumorigenesis, it is possible that the truncated isoform i_21_VEGFR-1 could be integrated into this circuit.

Another interesting question is the relationship between the truncated isoforms and cell stemness. The truncated intracellular KIT is expressed in hematopoietic stem cells and multipotent progenitors, but not in more differentiated cells. The c-KIT receptor and its ligand stem cell factor play an important role for the maintenance and differentiation of hematopoietic stem cells and multipotent progenitors [1,2]. Besides c-KIT, murine hematopoietic stem cells and multipotent progenitors also express the truncated intracellular form of the c-KIT receptor [35]. In contrast to c-KIT, whose expression is more widespread during murine hematopoiesis, tr-KIT expression is restricted to cell populations enriched for hematopoietic stem cells and multipotent progenitors. The truncated transcript and protein were downregulated when differentiation of primitive hematopoietic cells was induced with cytokines and retinoic acid. Interestingly, similar to a previous observation in mouse spermatogenesis [37], in hematopoietic cells, tr-KIT is phosphorylated at the C-terminal tyrosine Y161, through an as yet unidentified process. Expression of the truncated, ligand independent, isoform could play a specific role in hematopoietic stem and pluripotent progenitors.

## 10. Conclusions

There is still much work to be done to understand the biological and pathological functions of the truncated intracellular isoforms. However, the implication of i_21_VEGFR-1 in Src activation and the relevance of Src activation driving transformation, invasion, tumorigenesis and inflammation, could make the expression of i_21_VEGFR-1 an interesting therapeutic target for cancer and inflammation. Reversible proteasome inhibitors have emerged as a promising approach to the treatment of cancer and inflammatory diseases. We have shown that both the expression of VEGFR-1 and i_21_VEGFR-1 is downregulated by the reversible proteasome inhibitor MG262 [86]. More recently [24], we have studied the effect of a γ-secretase inhibitor, retinoic acid and the micro RNA miR-200c in the expression of i_21_VEGFR-1. As we have previously indicated, all these mechanisms are able to inhibit the expression of the VEGFR-1 intracellular truncated isoform. Particularly effective is the combination of the γ-secretase inhibitor and retinoic acid that almost completely abolishes the expression of the isoform. Both γ-secretase inhibitors and retinoic acid are being studied as potential therapies for breast cancer [80,87].
